# Demonstration of large ionization coefficient ratio in AlAs_0.56_Sb_0.44_ lattice matched to InP

**DOI:** 10.1038/s41598-018-27507-w

**Published:** 2018-06-14

**Authors:** Xin Yi, Shiyu Xie, Baolai Liang, Leh Woon Lim, Xinxin Zhou, Mukul C. Debnath, Diana L. Huffaker, Chee Hing Tan, John. P. R. David

**Affiliations:** 1 Department of Electronic and Electrical Engineering, University of Sheffield, Sheffield, S1 3JD UK; 2 School of Physics and Astronomy, Cardiff University, Cardiff, CF24 3AA UK; 3 California NanoSystems Institute, University of California-Los Angeles, Los Angeles, CA 90095 USA

## Abstract

The electron and hole avalanche multiplication characteristics have been measured in bulk AlAs_0.56_Sb_0.44_ p-i-n and n-i-p homojunction diodes, lattice matched to InP, with nominal avalanche region thicknesses of ~0.6 μm, 1.0 μm and 1.5 μm. From these and data from two much thinner devices, the bulk electron and hole impact ionization coefficients (α and β respectively), have been determined over an electric-field range from 220–1250 kV/cm for α and from 360–1250 kV/cm for β for the first time. The α/β ratio is found to vary from 1000 to 2 over this field range, making it the first report of a wide band-gap III-V semiconductor with ionization coefficient ratios similar to or larger than that observed in silicon.

## Introduction

Semiconductor based avalanche photodiodes (APDs) are widely used in long haul optical communication systems to increase the sensitivity of high speed receivers. Such APDs are required to detect signals at 1300 nm and/or 1550 nm which necessitates the use of a narrow band-gap semiconductor to detect the light. When subject to a large electric-field, the electrons and holes can gain sufficient energy to undergo impact ionization, increasing the number of carriers and thereby providing internal gain. Unfortunately, this large electric field can cause high tunnelling dark currents in narrow band-gap semiconductors and the stochastic nature of the impact ionization process can give rise to a high associated ‘excess’ noise which mitigates the effect of the gain. The first of these problems can be alleviated by the use of a low field narrow band-gap semiconductor to detect the long wavelength photons and a wider band-gap semiconductor at high electric-fields where the impact ionization occurs, the so called SAM-APD^1^. The two semiconductor materials need ideally the same lattice parameter enabling them to be grown monolithically without any strain occurring at the interface and this has resulted in almost all commercial APDs for telecommunication applications being based on InP substrates with InGaAs absorption regions^2,3^. In order to minimise the excess noise of the avalanche process, the electron and hole impact ionization coefficients (α and β respectively) need to be very different with the carrier having the larger ionization coefficient initiating the avalanche multiplication process^4^. Silicon has α/β ratios of >100 at electric fields of about 150 kV/cm^5^, ensuring extremely low excess noise performance and making them ideal for most applications in the visible to 1100 nm wavelengths. Unfortunately the quest to find a semiconductor material that has ‘silicon like’ disparate ionization coefficients and that is also lattice matched to InP is still ongoing. Traditionally, APDs for telecommunication applications have utilised InP as the avalanche gain medium, where the α/β ratio is 0.5 and holes have to initiate the avalanche multiplication process^6–8^ to ensure low excess noise. More recently AlInAs, lattice matched to InP has been gaining popularity as the α/β ratio is larger at 3 for a similar thickness of avalanching structure with electrons required to initiate the avalanche process^9,10^. While a material such as InAs^11^ has a very large α/β ratio, it also has a very narrow band-gap and cannot be grown lattice matched to InP. Recent publications have suggested that the AlInAsSb^12,13^ alloy also may have a large α/β ratio (~50–100), based on the evidence of very low excess noise measurements in thick avalanching structures. This alloy has a relatively large band-gap (at the higher aluminium mole fractions) with low dark currents at room temperature but it needs to be grown on more expensive GaSb substrates.

No material so far has provided us with the ideal combination of lattice matching to InP, low dark currents at room temperature and a very disparate ionization coefficient ratio. AlAs_0.56_Sb_0.44_ (hereafter referred to as AlAsSb) has a relatively large indirect band-gap of ~1.65 eV and is lattice matched to InP. There have been reports of very low excess noise performance in submicron AlAsSb p-i-n diodes^14^, but this is most likely due to the well known effect of the dead-space^15–17^ which can become significant in thin avalanching regions as wavelength dependent multiplication indicated that α/β~1 in these structures. An accurate determination of the α and β in this material ideally requires pure electron and hole initiated multiplication characteristics from p-i-n and n-i-p diodes with a range of avalanching widths where the effect of the dead-space is likely to be relatively insignificant.

In this work, we report for the first time the α and β in AlAsSb covering a wide range of electric fields. These α and β are not only capable of fitting the multiplication characteristics in our devices with ~660 nm-1550 nm thick p-i-n and n-i-p structures but also structures with avalanching widths as thin as 230 nm^14^.

## Results

A series of p-i-n and n-i-p AlAsSb photodiode structures (P1-P3, N1-N2) were grown by solid-source molecular beam epitaxy (MBE) lattice matched to InP substrates using a digital alloy growth technique and then fabricated into mesa diodes by wet chemical etching. The details of the layer thicknesses are given in Table 1 and P4, P5 refer to thin avalanching layers investigated previously. The growth and the fabrication details are given in the Methods section.

**Table 1:** Layer details.

Layer name	Diode type	Nominal i-thickness (μm)	Breakdown voltage V_bd_ (V)		CV Modelled results	
			Measured	Modelled fit	i-region doping (×10^15^ cm^−3^)	i-region thickness w (μm)
P1	PIN	1.5	84.5	85.7	5	1.55
N1	NIP	1.5	84.5	86.0	5	1.55
P2	PIN	0.60	40.2	40.4	10	0.66
N2	NIP	0.60	41.5	42.3	10	0.66
P3	PIN	1.00	62.2	63.3	10	1.15
P4^14^	PIN	0.25	20.2	21.2	1	0.23
P5^14^	PIN	0.10	11.2	14.9	1	0.08

**Figure 1:**
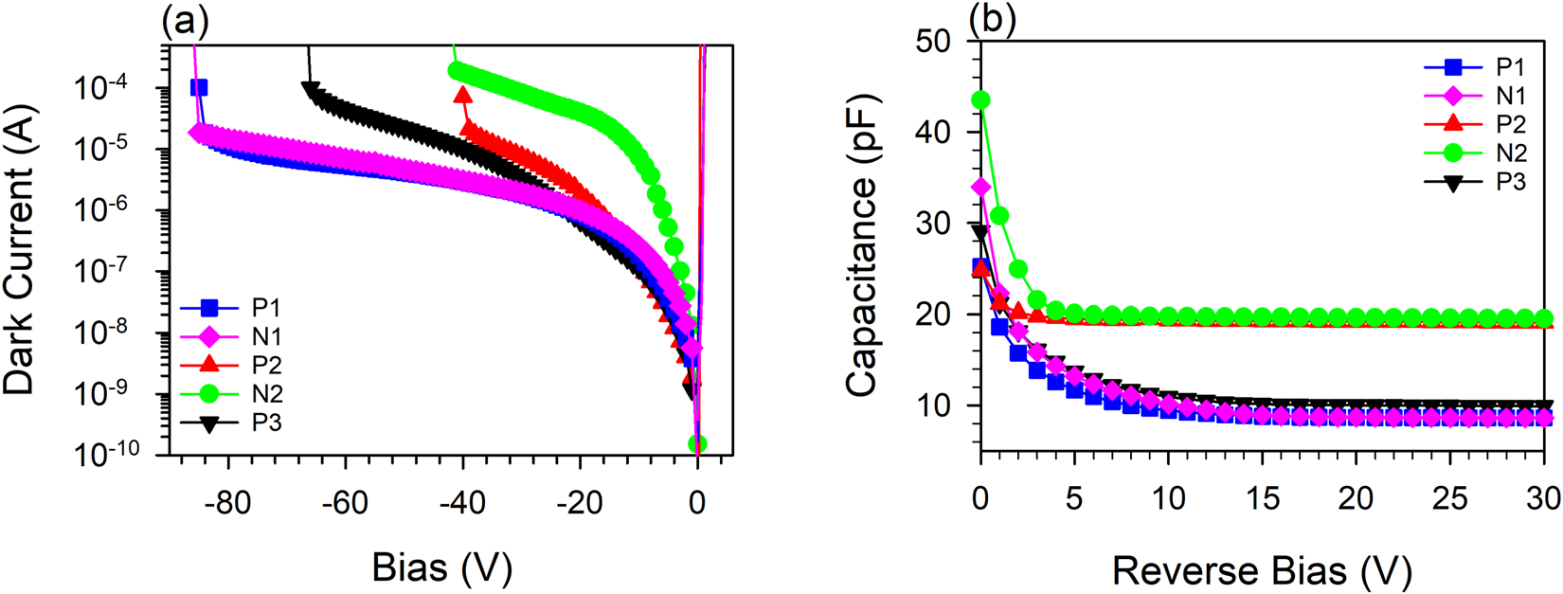
(**a**) Typical room temperature forward and reverse dark current-voltage characteristics. (**b**) Capacitance measurements as a function of reverse bias at room temperature for 420 μm diameter devices.

The forward and reverse bias currents in these devices were measured in the dark and are shown in Fig. 1(a). At low forward bias, the dark currents scaled with device perimeter suggesting the presence of large surface currents while at higher forward biases they scaled with area with ideality factors of ~2. The high reverse dark currents seen here are not indicative of the bulk dark currents in this material system as they too appear to scale with perimeter. In all the structures, a clear breakdown voltage could be seen.

The capacitance as a function of reverse bias for all the layers is shown in Fig. 1(b). After an initial rapid decrease in capacitance with bias, the capacitance remains approximately constant suggesting that the intrinsic region is fully depleted and there is negligible depletion in the heavily doped cladding regions. The depletion widths and doping levels obtained from these measurements are summarized in Table 1. The doping density varies between 5 × 10^15^ – 10 × 10^15^ cm^−3^ resulting in a tapered electric-field profile in the intrinsic region (shown in Fig. 1 in Supplementary Information).

The photocurrent versus reverse bias was obtained using light at 405- and 633-nm wavelength lasers, focussed onto the top of the 420 μm diameter devices (shown in Fig. 2 in Supplementary Information). Due to the relatively high surface dark currents, the photocurrent measurements were undertaken by modulating the laser light and using phase sensitive detection techniques. The 405-nm light is assumed to be almost fully absorbed in the top doped cladding layers so will provide single carrier injection into the avalanching region (giving us M_e_ on the p-i-n’s or M_h_ on the n-i-p’s) while the 633 nm light should only be weakly absorbed and will generate carriers uniformly throughout the structure, providing a ‘mixed’ injection multiplication characteristic (M_mix_). The determination of the absorption coefficients at these wavelengths is given in the Methods section. Measurements were undertaken on at least 3 different devices for each structure at different laser powers to ensure the repeatability of results. The multiplication in these structures was determined by looking at the increase in photocurrent once the intrinsic layer was fully depleted and correcting for any increase in the primary photocurrent due to small movement of the depletion edges in the heavily doped cladding regions^18^.

**Figure 2:**
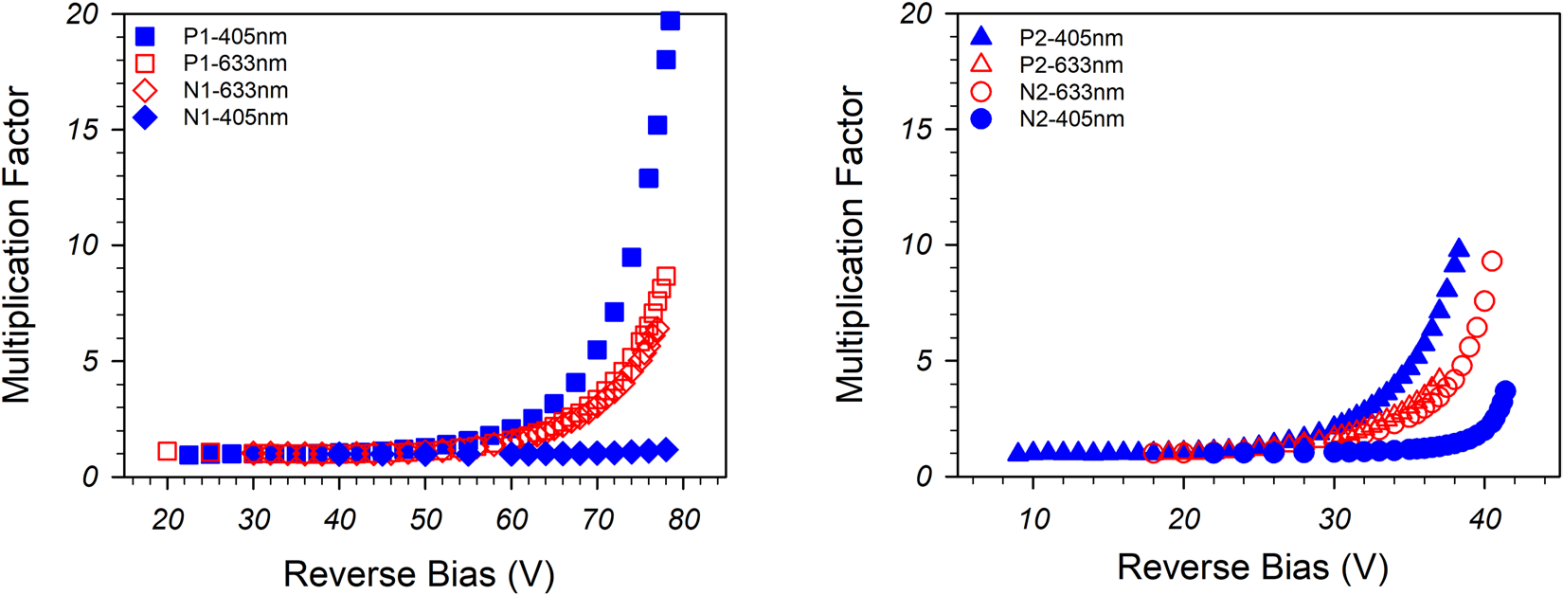
Multiplication factor versus reverse bias of (**a**) P1 and N1, (**b**) P2 and N2.

Figure 2 shows the multiplication characteristics from these structures and indicates that α > β in this material because the M_mix_ ≤ M_e_ in the p-i-n structures and M_mix_ ≥ M_h_ in the n-i-p structures. The M_mix_ results (obtained using 633 nm illumination) for P1 and N1, and P2 and N2 are very similar as each pair has very similar intrinsic layer thicknesses and doping profiles. The M_e_ from the three p-i-n structures are shown on a log plot in Fig. 4 in Supplementary information.

## Discussion

The ionization coefficients as a function of electric-field were extracted from the multiplication results of P1-5 and N1-2 taking into consideration any tapered electric-field profile but ignoring any dead-space effects^19^ by using a trial and error fitting technique which minimised the difference between the experimental and modelled data. The parameterized ionization coefficients cover a wide electric field range from 220–1250 kV/cm for α and from 360–1250 kV/cm for β and are given by;

1 $$\alpha (E)=5.5\times {10}^{5}\exp (-{(\frac{12.1\times {10}^{5}}{E})}^{1.43}){{\rm{cm}}}^{-1}$$

for 220 KV/cm ≤ E ≤ 500 KV/cm and

2 $$\alpha (E)=4.0\times {10}^{5}\exp (-{(\frac{12.5\times {10}^{5}}{E})}^{1.25}){{\rm{cm}}}^{-1}$$

for 500 KV/cm < E ≤ 1250 KV/cm,

3 $$\beta (E)=3.2\times {10}^{5}\exp (-{(\frac{17\times {10}^{5}}{E})}^{1.6}){{\rm{cm}}}^{-1}$$

for 360 KV/cm ≤ E ≤ 1250 KV/cm.

**Figure 3:**
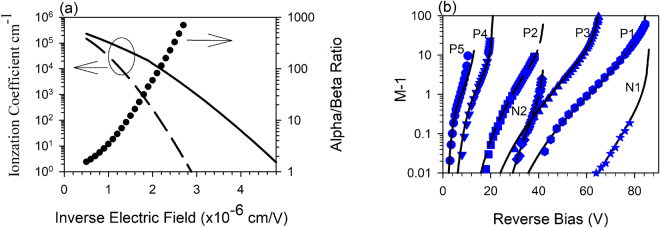
(**a**) Ionization coefficients α (Solid line) and β (Dashed line) and α/β ratio (Symbols) of AlAsSb. (**b**) Experimentally obtained M-1 (Symbols) and modelled (Lines) for P5, P4, P2, N2, P3, P1 and N1, going from left to right.

These ionization coefficients are shown graphically in Fig. 3(a), as is the α/β ratio as a function of inverse electric-field. These coefficients are capable of reproducing the measured M_e_ and M_h_ for the different avalanche layer structures accurately as shown by the plot of (M-1) on a logarithmic scale in Fig. 3(b). Excellent agreement was achieved even at very low gain values of M~1.01. Figure 3(a) shows that an α/β ratio of >100 can be achieved at electric fields below 460 kV/cm and this ratio can exceed 1000 at electric fields below 360 kV/cm. The onset of measurable M_e_ occurs well below the breakdown voltage in P1-P3, in contrast to the M_h_ in N1 and N2 as shown in Fig. 3(b). This is because in our samples we can accurately measure multiplication values down to 1.01 and at the electric-field where M_e_ gives us 1.01, the value of β is too low to give any measurable M_h_. Although the M_h_ could only be experimentally obtained over a limited range in N1, the good agreement with the model down to values of M_h_ = 1.01 suggests that the low electric-field data for β is correct.

These coefficients can also replicate the M_e_ in P4 reasonably well if we use the parameters given in Table 1 but the fit to M_e_ in P5 is slightly worse at higher values of multiplication, even when allowing for the depletion into the p^+^ and n^+^ cladding regions. The reason for this is likely to be due to the fact that at very high electric fields in very thin structures, the ionization coefficients are not simple functions of electric-field^20^ and more sophisticated modelling techniques are required to fit to the experimental data.

**Figure 4:**
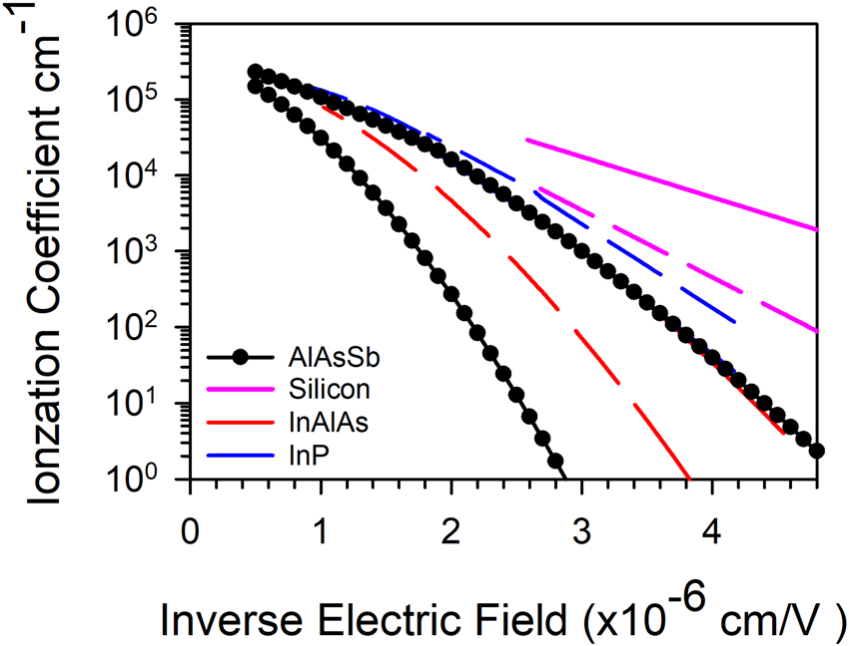
Ionization coefficients α (Solid line) and β (Dashed line) of AlAsSb compared to InAlAs [10], InP [6] and silicon [5]. α of InAlAs and InP are almost identical to that of AlAsSb and are difficult to separate in the figure.

To further emphasis how much larger the α/β ratio in AlAsSb is compared to normal III-V materials and silicon, Fig. 4 presents the ionization coefficients of AlAsSb, together with those of InP, InAlAs and silicon as a function of inverse electric field. AlAsSb can be seen to have a significantly larger α/β ratio so this should give rise to APDs with very low excess noise. For example, an ideal 1 μm p-i-n structure operating at a gain of 10, would have an α/β ratio of >40.

The rapidly increasing α/β ratio as the electric field decreases also explains the unusual result observed by Xie *et al*.^14^, whereby the excess noise decreased significantly as the avalanche thickness increased from 80 nm to 230 nm, contrary to results observed in InAlAs^21^ and Si^22^. The α in all three III-V avalanche materials shown in Fig. 4 appear to be almost identical, despite quite different conduction band structures but the most interesting observation is that the β is significantly lower in AlAsSb, giving rise to the large α/β ratio.

In summary, the avalanche multiplication characteristics of AlAsSb have been measured in a series of diodes with avalanche region widths from 1.55 μm down to ~80 nm. From these, the ionization coefficients as a function of electric-field have been extracted and the α/β ratio is found to much larger than any other wide band-gap III-V semiconductor material with values of >100 for electric fields below 460 kV/cm. This makes AlAsSb ideal for the multiplication region in InP based telecommunication SAM-APDs.

## Methods

The AlAsSb p-i-n and n-i-p structures are grown on epi-ready n- InP (001) and p-InP (001) substrates, respectively, via digital alloy growth technology in a Veeco GEN930 MBE reactor, in which both As_2_ and Sb_2_ fluxes are supplied using valved cracker cells. Due to the wide miscibility band gap, growth of thick high-quality and lattice-matched AlAsSb alloy is extremely challenging because of the non-unity sticking coefficient of group-V species^23,24^. Both fast fluctuations and slow drifts in group-V cell temperatures can also result in composition fluctuations and departure from the exact lattice-matched condition. In order to get the precise lattice-matched AlAsSb alloy with high crystalline quality, digitally grown AlAsSb is realized by periodically alternating the As and Sb shutter to obtain the desired alloy composition. The details of the growth of lattice-matched digital alloy AlAsSb on InP (001) [used for this study] and GaSb (001) substrates are given elsewhere^25,26^.

**Figure 5:**
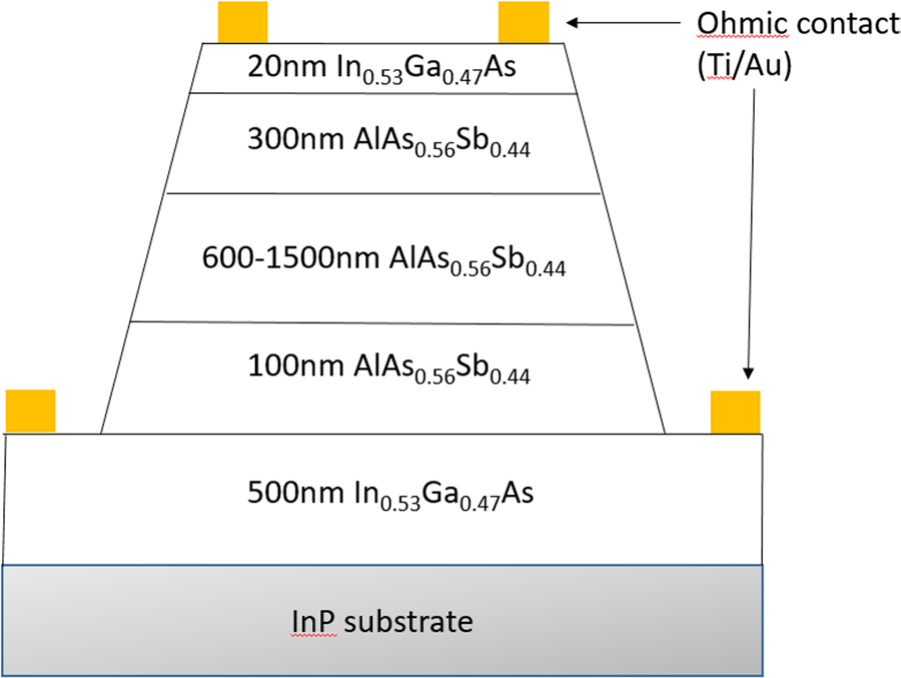
Diagram of an AlAsSb mesa device.

A schematic diagram of the device structure is shown in Fig. 5. The i-region is sandwiched between a 300-nm p^+^/n^+^ AlAsSb and a 100-nm p^+^/n^+^ AlAsSb cladding layers with Be and Te doping concentrations of 2.0 × 10^18^ cm^−3^. The structure also has a highly doped (1.0 × 10^19^ cm^−3^) top 20-nm and bottom 500-nm InGaAs contact layers. Mesa devices were fabricated by standard lithography and wet chemical etching where a 2: 1 mixture of citric acid (1 g citric acid powder to 1 ml of de-ionised water (DIW)) and hydrogen peroxide (H_2_O_2_) is used for the removal of the InGaAs cap layer and a 1: 2: 10 mixture of hydrochloric acid, diluted H_2_O_2_ (with a ratio of 1 part peroxide to 9 parts DIW) and DIW to etch the AlAsSb layer into circular mesa diodes with diameters of 70, 120, 220, and 420 μm. Ti-Au was used to form the top and bottom metal contacts.

A HP4275A LCR meter was used to perform capacitance-voltage measurements at a frequency of 1 MHz. A static dielectric constant, ${\varepsilon }_{r}$, of 10.95 was used to determine the depletion widths and background doping concentration. Dark current-voltage measurements were undertaken using a HP4140B picoammeter and a probe station.

The multiplication characteristics, M, as a function of reverse bias were determined from modulated photocurrent measurements obtained using a lock-in amplifier with phase-sensitive detection to remove the dc leakage currents. The impact ionization coefficients were determined by fitting to the experimentally determined multiplication data using the equation below^27^ with the doping density and i-region thickness as shown in Table 1.

4 $$M({x}_{0})=\frac{\exp \{-\,{\int }_{0}^{{x}_{0}}[\alpha ({x}^{^{\prime} })-\beta ({x}^{^{\prime} })]d{x}^{^{\prime} }\}}{1-{\int }_{0}^{W}\alpha ({x}^{^{\prime} })\exp \{\,-\,{\int }_{0}^{{x}_{0}}[\alpha ({x}^{^{\prime} })-\beta ({x}^{^{\prime} })]d{x}^{^{\prime} }\}dx}$$

Different wavelength light was used to generate pure carrier or mixed carrier multiplication in the diodes. As no published absorption coefficient of AlAsSb is available, we linearly interpolate between the absorption coefficients of AlAs and AlSb^28,29^ to obtain values of 2.0 × 10^5^ cm^−1^ at 405 nm and 3.6 × 10^2^ cm^−1^ at 633 nm. Due to the large InGaAs/AlAsSb conduction band offset^30,31^ carriers created in the top 20 nm InGaAs cap layer do not contribute to the photocurrent.

## Electronic supplementary material

Supplementary Information
