# Supplementary material for: Demonstration of large ionization coefficient ratio in AlAs0.56Sb0.44 lattice matched to InP
Source: Sci Rep. 2018 Jun 14;8:9107. doi: 10.1038/s41598-018-27507-w (PMC6002549; doi:10.1038/s41598-018-27507-w)
Supplement: Supplementary file 1 — Supplementary Information [file 41598_2018_27507_MOESM1_ESM.docx]

**Supplementary Information**

**Demonstration of large ionization coefficient ratio in AlAs_0.56_Sb_0.44_ lattice matched to InP**

Xin Yi^1^, Shiyu Xie^2^, Baolai Liang^3^, Leh Woon Lim^1^, Xinxin Zhou^1^, Mukul C. Debnath^3^, Diana L. Huffaker^2^, Chee Hing Tan^1^, and John. P. R. David^1^.

^1^Department of Electronic and Electrical Engineering, University of Sheffield, Sheffield S1 3JD, UK.

^2^School of Physics and Astronomy, Cardiff University, Cardiff CF24 3AA.

^3^California NanoSystems Institute, University of California-Los Angeles, Los Angeles, CA 90095, USA.

Correspondence should be addressed to S.Y.X. (email: XieS1@cardiff.ac.uk) and J.P.R.D. (email: j.p.david@sheffield.ac.uk)





Figure 1. The intrinsic region doping density.

Figure 1 shows that the intrinsic region doping density for P2, P3 and P1 varies between 5 × 10^15^ – 10 × 10^15^ cm^-3^ as determined from the C-V measurements.





Figure 2. The photocurrent versus reverse bias using 405-nm and 633-nm laser illumination.

Figure 2 shows the photocurrent versus reverse bias obtained using light from 405- and 633-nm wavelength lasers, focussed onto the top of the 420 μm diameter devices. These are shown with different laser powers thereby vertically offsetting the results for clarity. The background doping in the intrinsic regions is found to be p-type from the bias dependence of the spectral response in these layers, as shown in Figure 3. The initial rapid increase in photocurrent with bias at short wavelengths in p-i-n layers is due to the rapid change in carrier generation profiles with distance and the movement of the depletion edge towards the top p^+^ layer. No such behaviour is seen at short wavelengths in the n-i-p structures as the depletion edge moves towards the bottom p^+^ layer. In both the p-i-n and n-i-p structures, the increase in photocurrent with bias at the longer wavelengths (where the carrier generation profile is almost constant with distance) can be attributed primarily to absorption in the depletion region, whose width increases with reverse bias as shown by the C-V measurements. Once the device is fully depleted, the photocurrent remains relatively constant with further increases in reverse bias until impact ionization occurs.







(b)

(a)

Figure 3. Measured spectral responses on P2 (a) and on N2 (b).

A 100 w tungsten bulb and a grating monochromator were used to measure the spectral response of each layer at different biases. Figure 3 (a) and (b) present the measured photocurrent of P2 and N2 with increasing reverse bias from 0-8 V. The photocurrent at shorter wavelengths (<450 nm) in the p-i-n structure increases more rapidly between 0-8 V than in the case of the n-i-p, where the photocurrent appears to be constant over this range. This suggests that the background doping in the intrinsic regions is p-type. After 8 V, the i-region is fully depleted as shown by the CV measurements. Similar behaviour was observed in the other layers, depending on whether it is a p-i-n or n-i-p.





Figure 4. The M-1 versus reverse bias using 405-nm laser illumination.

Figure 4 shows the M_e_ obtained using 405nm illumination on the three p-i-n diodes, plotted on a log scale. The shape of the curves with the onset of multiplication occurring well before the breakdown voltage is expected when α>>β.
